# The Sphygmograph

**Published:** 1866-11

**Authors:** 


### *The Sphygmograph.*

The Sphygmograph, which is exciting some attention just now, is one of those ingenious instruments which have been devised of late years, promising really to assist medical men in reducing their art to something like a science. Its great merit is this: that it gives a permanent and minutely accurate record of a phenomenon which before was known only by the very unsatisfactory discrimination of the sense of touch.

The sphygmograph is an instrument for producing a self-written record of the swellings and contractions of the arteries known as the pulse.

Its inventor, Dr. J. E. Marey, is a Paris physician, who is well known for many physiological essays. The main features presented by the instrument are the following:

A principal beam of light construction is fastened on the arm by padded straps; to this is attached a lever of nearly the length of the forearm; the shorter arm of this lever rests gently, but firmly, on the pulse; at each rise of the artery, and subsequent fall, the motion is exactly imparted to the lever, and the end of the longer arm performs the same movements as does the shorter, but on a much larger scale. To the end of the longer arm is attached a fine pointed pencil, in contact with which a smooth strip of paper is made to move by clockwork in a horizontal direction. The effect of this arrangement is, that a straight line would be drawn on the piece of paper were it not for the rhythmic perpendicular movement caused by the pulse, which results in the production of an undulated line, the waves in which represent the separate expansions of the artery; of course, it is evident that since the movement of the paper is invariably uniform, the variations in the pulse will be distinctly indicated by the height, length, and form of the waves; and accordingly we have a most accurate and valuable means of comparing the pulse in various individuals and under various circumstances. Some interesting results have been obtained by studying the pulses of diseased persons, and the instrument has been found to exhibit phenomena in the pulse which it was quite impossible to detect by the rough-and-ready means of the fingers. The "sphygmogram" of a person afflicted with a certain disease of the heart, for example, is found to exhibit a series of undulations, the ascending line of which is very long and tremulous, and but slightly oblique, while the descending is abrupt and nearly perpendicular. The application and value of the instrument will be apparent from these remarks.—*Quarterly Journal of Science*, *London*, *July*, 1866.

